# Uniportal VATS technique for primary spontaneous pneumothorax: An analysis of 46 cases

**DOI:** 10.12669/pjms.36.2.1556

**Published:** 2020

**Authors:** Hasan Oguz Kapicibasi

**Affiliations:** Hasan Oguz Kapicibasi, MD. Department of Thoracic Surgery, Canakkale Onsekiz Mart University, Faculty of Medicine, Canakkale, Turkey

**Keywords:** Pleurectomy, Primary spontaneous pneumothorax, Uniportal, VATS

## Abstract

**Objective:**

In the treatment of primary spontaneous pneumothorax (PSP), the influence of safety and applicability of uniportal video assisted thoracoscopic surgery (VATS) bullectomy/blebectomy and pleurectomy method were evaluated in 46 patients.

**Methods:**

Between November 2010 and January 2019, 46 patients (36 males, 10 females; mean age 24.2 years; range 16-36 years) undergoing uniportal video thoracoscopic bullectomy/blebectomy and apical pleurectomy for primary spontaneous pneumothorax were evaluated retrospectively at Canakkale Onsekiz Mart University (COMU). One patient underwent surgery for the second time after three months for contralateral pneumothorax and counted as two different patients, hence forty seven operations were performed in total. The cases were evaluated in terms of age, gender, comorbidity, duration of hospital stay, histopathological diagnosis, postoperative morbidity and mortality.

**Results:**

Right surgical intervention was performed in 20 cases (42.6%) and left surgical intervention in 27 cases (57.6%). A total of 15 (31.9%) surgical operations were performed during the first attack, 30 (63.8%) during the second attack and 2 (4.3%) during third and more attacks. There was prolonged air leakage in all patients operated during the first episode. All cases underwent wedge resection and pleurectomy with endoscopic stapes. None of the patients required thoracotomy. Postoperative drainage period was between 2-7 days (mean: 4.1) and the duration of hospitalization was between three to eight days. Postoperative pain and paraesthesia were observed in eight cases. Prolonged air leakage was observed in five cases.

**Conclusion:**

With video thoracoscopic uniportal technique, not only lung biopsy and resection but also bullectomy/blebectomy and pleurectomy operations can be performed safely in the treatment of PSP. In view of this information, minimally invasive techniques are seen as more advantageous than conventional techniques.

## INTRODUCTION

Spontaneous pneumothorax is more common in adult males than females. The incidence of primary spontaneous pneumothorax in young men per year is 7.4-18 / 100.000 and in young women, this rate is 1.2-6 / 100.000. On the other hand, secondary spontaneous pneumothorax is a similar feature in males to 6.3/100.000 and 2/100.000 in females. In children, it is estimated that there are 4 / 100,000 in boys and 1.1/100,000 in girls each year. Other risk factors include smoking, having a weak and long body structure.^1^,^2^ As stated in the British Society of Thoracic Surgeon (BTS) guidelines in the treatment of primary spontaneous pneumothorax, the first relapse in the treatment of pneumothorax should be pleural adhesions with bullectomy.^3^ Recently, VATS (video-assisted thoracic surgery) is considered the gold standard for the treatment of pneumothorax.^4^ The VATS approach has been shown to offer greater advantages in patient pain and respiratory function compared to thoracotomy incisions. The standard multi-port approach has replaced the single-port single incision or uniportal approach over time. Uniportal approach has been shown to be safe and effective in lobectomy with lung resections and biopsies. On the PSP, bullectomy / blebectomy are used safely in pleural abrasion/pleurectomy by placing a single incision drain.^5^

In this study, we retrospectively evaluated the efficacy and applicability of uniportal video-assisted thoracic surgery operations (by the author, most of whom are in the second level state hospital) increasingly preferred all over the world in PSP.

## METHODS

Between November 2010 and January 2019, 46 patients who underwent uniportal video thoracoscopic blebectomy / bullectomy and apical pleurectomy because of primary spontaneous pneumothorax were evaluated retrospectively at Izmit Seka State Hospital and COMU Medical Faculty. Patients with previously diagnosed lung disease were excluded from the study. The study was approved by the Ethics Committee of Çanakkale Onsekiz Mart University (Date of meeting: 22.05.2019 - Decision No: 2019/11–01).

One of the cases was evaluated as two separate cases because of contralateral pneumothorax after three months for applying the surgical procedure on the opposite side. The cases were evaluated in terms of age, gender, comorbidity, histopathological diagnosis, postoperative morbidity and mortality. Smokers were considered smokers who smoked at least once a day. Thorax computed tomography (CT), electrocardiography, complete blood count, basic biochemical tests (sodium, potassium, chloride, bicarbonate, blood urea nitrogen-BUN, magnesium, creatinine, glucose) was performed to determine in PA / L (posteroanterior / lateral) chest X-ray, bulla and bleb number and placement in all patients before the operation. In his anamnesis, the cases that did not have any lung disease with clinical radiology and laboratory findings were evaluated as primary spontaneous pneumothorax and were included in the study. Demographic data, as well as postoperative hospital stay, recurrence rates, and other complications, were also evaluated. Recurrent ipsilateral pneumothorax, contralateral pneumothorax and long air leakage of more than five days and the bulla detected in PA lung radiography were considered as surgical indications.^4^,^6^ The approach with uniportal VATS in the treatment of PSP has the same advantages as the traditional three-part approach. The approach with VATS enables the exploration of the thoracic cavity, the detection of bulla bleb in the lung parenchyma, the determination of lung diseases and application of pleurodesis (mechanical parietal pleural abrasion, talcum powder, pleurectomy, etc.).^7^ All patients were intubated with the double-lumen tube under general anesthesia and lateral decubitus position, the operation was performed using 10 mm 30-degree endoscopy, endoscopic grasper and endoscopic linear stapler from the midaxillary line five intercostal space. The adhesions between the pleural leaves were removed by blunt and sharp dissection. Bulla/bleb was resected with an endoscopic linear stapler. Parietal pleurectomy was performed with the help of thoracoscopic forceps up to the 7th ıntercostal space from the apical pleural region. All patients had the same surgical procedure, consisting of bullectomy and pleurectomy. Obtained pleurectomy material was sent to histopathologic examination with lung wedge resection material. At the end of procedure, 28 F polyethylene drain was placed in all patient and the lung was re-expanded with 30 mm Hg pressure. The chest tube was then removed the day after air-leak stopped and after chest X-ray demonstrated a well expanded lung.

### Statistical analysis

Clinical findings, pathologic diagnoses and sociodemographic qualities of the patients who were operated in our clinic, were given in numbers and percentages as descriptive data.

## RESULTS

The mean age of 46 patients who underwent Uniportal VATS bullectomy/blebectomy and pleurectomy was 24.1 (16-36), 10 (21%) females and 36 (79%) males. Comorbidities of all patients recorded (Table-I).

**Table-I:** Comorbidities.

Comorbidity	No (%) of patients
Obesity	2 (4)
Diabetes Mellitus	1 (2)
Hypertension	1 (2)
Scoliosis	1 (2)
Wilson Disease	1 (2)
Familial Mediterranean Fever?	1 (2)
Herpes Zoster Infection	1 (2)

Total 15 (31.9%) surgical operations were performed during the first attack, 30 (63.8%) during the second attack and 2 (4.3%) during third and more attacks. All patients who underwent surgery during the first attack had prolonged air leakage. Of the cases, 23 were active smokers 13 of them used it at some time in their lives and 10 had not smoked at any time.

**Fig.1a:**
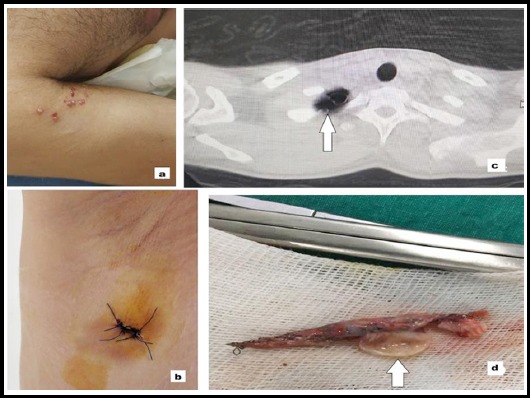
Varicella zoster skin lesion, **1b:** The uniportal incision is made on the 5th intercostals space, anterior axillary line. (2,5 cm), **1c:** CT scan of the chest showing bullae (arrows) in the apex of the lung, **1d:** Thoracoscopic resection for bleb disease.

A young male patient in this group had rare varicella zoster virus pneumothorax coexistence and was operated for prolonged air leakage (Fig. 1a, 1b, 1c, 1d).

Previously, a young female patient with recurrent bilateral pneumothorax was misdiagnosed. The patient was diagnosed as familial familial mediterranean fever and treated for two years. After the bilateral operation of the patient the drugs were cut by consulting the advanced center. The other young female patient who was diagnosed with Wilson disease was discharged on the second postoperative day. Postoperative pain-paresthesia and prolonged air leakage were the most common complications (Table-II).

**Table-II:** Complication.

Complication	No (%) of patients
Prolonged Air Leakage	5 (10)
Postoperative pain-paraesthesia	8 (17)
Recurrence	1 (2.1)
Atrial fibrillation	1 (2.1)
Subcutaneous emphysema	1 (2.1)

Thoracotomy was not required in any of our patients. Air leakage was detected in 2 (4%) cases where a parenchymal lesion could not be detected and wedge resection was applied with endostaps such as other cases. Postoperative drainage period was between 2-7 days (mean: 4.1) days and the duration of hospital stay was 3-8 days. The mean blood loss was 100 ml or less in all cases. No mortality was observed in any of our cases. Histopathological results were reported as chronic pleuritis and there was no correlation between pathological outcome and recurrence. In a patient who was discharged, a partial pneumothorax was observed on the 14th postoperative day. The patient who did not accept the thoracotomy was discharged after a closed tube underwater drainage system with 28 F polyethylene drain and tube thoracotomy on the 5th day. No recurrence was found in the subsequent follow-up of the cases.

## DISCUSSION

The goals of surgical treatment in PSP are the prevention of air leakage and recurrence of the disease.^8^ Many authors believe that, watchful waiting, aspiration or tube thorocostomy is sufficient in first spontaneous pneumothorax while they concluded that in the second attack surgery is necessary. In non-surgical treatments, the recurrence rate after the first attack is 25-30%, and the recurrence rate after the second and more attacks reaches 60-80%.^9^ In addition, the tension pneumothorax and the history of hemopneumothorax are among the goals of surgical treatment to prevent recurrence for high-risk occupations such as divers and pilots.^10^

In order to prevent recurrence, the majority of surgeons prefer blebectomy / bullectomy and parietal pleurectomy or abrasion methods and use thoracotomy, multiport VATS and finally uniport VATS techniques.^11^ VATS has been developed over many years and has been the surgical treatment of PSP in the early period. With advances in thoracoscopic instruments and techniques, VATS blebectomy / bullectomy is still a preferred procedure for many centers in the treatment of PSP.^12^ In studies performed, uniportal VATS technique, bullectomy, and mechanical pleurodesis were found to provide a significant reduction in hospital stay, pain, and paresthesia.^5^ In a recent systematic review, the lowest recurrence rates after spontaneous pneumothorax treatment have been reported in wedge resection + mechanical pleurodesis + chemical pleurodesis group compared to other combined therapies.^13^ The uniportal approach, 30-degree thoracoscopy and the way to achieve success with the use of roticulator stapler,^14^,^15^ as stated in the studies with the surgical incision length of 2-4 cm in a shortened.^11^,^16^ In our study, the length of our incision was 2.5 cm (2-3 cm) and was parallel to the other studies. We believe that the rapid development of endoscopic surgical instruments will shorten the incision size. Furthermore, it can be concluded that uniportal VATS can reduce postoperative pain and paresthesia findings and uniportal VATS technique can be a safe, feasible and effective treatment for PSP.^17^ Horio et al. argued that patients operated with VATS had a higher recurrence rate than thoracotomies and although they were related to the presence of bulla/bleb which could not be detected by VATS,^18^ however Sawada et al. found no significant difference between the two groups.^19^ The postoperative recurrence rate in uniport VATS operations in primary spontaneous pneumothorax was found between 0-17.9%.^20^,^21^ In our study, the recurrence rate was 2.1%. In the studies performed, no mortality was seen in the surgical method^3^ and in our study, no mortality was observed. Prolonged air leakage after surgery is one of the most common complications and other morbidities reported in the literature: pneumonia, atelectasis, and need for mechanical ventilation, wound infections, empyema, pleural effusion, ARDS and Horner’s syndrome.^22^,^23^ Prolonged air leakage was reported to be 14.8% in studies.^24^ In our study, we observed prolonged air leakage in 5 (12.7%) patients. Postoperative chronic chest pain and paresthesia were reported in 21% of the patients,^25^ and this rate was 8 (17%) in our study. Only one patient who described the postoperative pain and paresthesia and who did not benefit from medical treatment was sent to the pain clinic and recovered completely during subsequent follow-up. A young female patient operated for bilateral pneumothorax was diagnosed with FMF because of the recurrent acute chest and reflected pain episodes. FMF lung involvement is quite rare. Fifty-one patients with lung amyloidosis were detected in the Mayo Clinic between 1980 and 1993, and only one patient had FMF and both had bilateral infiltration and pathological findings of adenopathy.^26^ In our patient, there was no radiological infiltration sign and adenopathy and no pathology of amyloidosis was observed. In the light of all these examinations, the patient’s medications were re-consulted and discontinued the advanced center immediately after the operation, and the patient’s complaints were attributed to recurrent pneumothorax attacks due to lack of old clinical findings. Another young male patient had a rare varicella zoster virus pneumonia secondary to pneumothorax^27^ is not observed recurrence during follow-up after the operation patients.

### Limitations of the study

The number of cases in our study is limited and this is a retrospective study. The uniportal VATS treatment of spontaneus pneumothorax, could not be compared with other surgical methods (Multiport VATS, axillary thoracotomy). Thereby, the advantages of uniportal VATS are not presented.

## CONCLUSION

Uniportal thoracoscopic approach used in the treatment of primary spontaneous pneumothorax, surgical instruments and rapid developments in surgical technique, thoracic surgery as a safe and effective method will continue to be in widespread use and will be preferred against conventional methods. With the low morbidity and mortality rate, it can be applied safely outside of the advanced centers.
